# Optimizing the Relationship between Regulation and Innovation in Dietary Supplements: A Case Study of Food with Function Claims in Japan

**DOI:** 10.3390/nu15020476

**Published:** 2023-01-16

**Authors:** Keigo Sato, Kota Kodama, Shintaro Sengoku

**Affiliations:** 1 Department of Innovation Science, School of Environment and Society, Tokyo Institute of Technology, Tokyo 108-0023, Japan; 2 Graduate School of Technology Management, Ritsumeikan University, Osaka 567-8570, Japan; 3 Life Style by Design Research Unit, Institute for Future Initiatives, The University of Tokyo, Tokyo 113-0033, Japan

**Keywords:** functional foods, dietary supplements, foods for specified health uses, foods with function claims, regulation, innovation

## Abstract

Regulation has long been a counterpart of innovation in the health care industry, and recent cases have demonstrated that appropriately designed regulations can both coexist with and promote innovation. This study is the first study to explore how the regulatory environment affected the innovation process during the transition of the regulations for functional foods in Japan by examining quantitatively the impact of the foods with function claims (FFC) system on industry, companies, and products. Based on a dataset of Japanese dietary supplement manufacturing companies (n = 169) and their products (n = 731) in 2019, we found that companies that have newly entered the FFC system are smaller in scale than existing companies (*p* < 0.01, Wilcoxon rank sum test). We also found that companies with FFC products have larger revenue growth (*p* = 0.01). A multiple regression analysis revealed that FFC product sales increased with in-house clinical testing (coefficient: 26.8, *p* < 0.0001), diverse active ingredients (coefficient: 7.6, *p* < 0.001), and the claim of new functions (coefficient: 10.2, *p* < 0.05). These results suggested that the FFC system facilitated the market entry of small and mid-size enterprises and promoted the creation of high-value products through innovative company efforts.

## 1. Introduction

Innovation in the health-care sector contributes to health promotion and disease prevention, especially chronic lifestyle-related diseases in aging populations, with the resultant reduction of public health-care costs [1]. In most industrialized countries, policy makers strictly regulate this sector to protect the safety and health of consumers. Although regulations can increase costs, restrict firms’ freedom of action, and hinder innovation well-designed regulations can induce investment in innovation, process implementation, and new product releases [2,3]. Thus, regulation has either positive or negative aspects for innovation depending on the characteristics of the business or the technology [4,5]. To promote innovation, policy makers must understand the multifaceted nature of regulations and design them appropriately to stimulate the market and benefit consumers.

Efficient regulation can help introduce innovation in the health-care sector [3]. With the lowering or removing of barriers to competition, deregulation often stimulates the market entry of new competitors with alternative technologies or business models [6]. For example, regulatory reforms implemented by the United States Food and Drug Administration (FDA) have driven the growth of FDA-approved mobile medical apps [4]. This suggests that regulatory health-care reform—when properly implemented—can stimulate innovation in technology and the delivery of health care.

While understanding and utilizing optimal regulation is important to promote innovation in a regulatory environment, there is little previous research discussing how regulations in the health-care sector, especially the functional foods sector, can stimulate innovation in technology, as discussed in following section. Although Japan has a large functional foods market of USD 20 billion per year, there is a lack of research describing the industrial structure and product properties in detail in the Japanese functional foods industry under the regulatory system. This paper is the first study to examine empirically and quantitatively the impact of the transition of the regulation of Japanese functional foods since 2015 on the dietary supplement industry, manufacturing companies, and the associated products. The study focuses on the change of the Japanese regulatory system for foods with health claims (FHC) as an opportunity to observe the influence of the regulations on innovation, and aims to obtain insights into the relationship between innovation and regulation.

We provide a literature review and our hypotheses in Section 2, the methodology of this study in Section 3, results in Section 4, discussion in Section 5, and conclusions in Section 6.

## 2. Literature Review and Hypotheses

### 2.1. Functional Foods: Industry Overview and Products

The concept of functional food has its origin in traditional Asian medicine and ancient texts and was proposed in Japan in the 1980s. In this paper, “functional foods” refer to the foods that offer health benefits beyond their nutritional value. Terms such as “functional foods” or “nutraceuticals” are widely used in the marketplace.

Such foods are regulated by the United States Food and Drug Administration (FDA) under the authority of the Federal Food, Drug, and Cosmetic Act, even though they are not specifically defined by law. In the US, functional ingredients added to foods to make them “functional foods” must be approved by the FDA as a food ingredient. In Japan, terms referring to “functional foods” or “dietary supplements” are not defined by law and no legal system exists to comprehensively regulate dietary supplements and functional foods. Some of them are covered by regulation systems for foods with health claims, as discussed below. Japanese regulations are attributed to the historical path-dependency of a repeatedly revised system [7].

Proponents of so-called functional foods (i.e., both unmodified foods and dietary supplements, also known as nutraceuticals or nutrition supplements) claim that these food products potentially promote health, mitigate lifestyle-related chronic diseases, and reduce public health-care costs. Driven partly by consumers’ desire to take a proactive approach to their health care, the global market for dietary supplements has steadily expanded along with increasing consumer acceptance and food technology innovation [8,9,10,11]. The dietary supplement industry incorporates various business entities, such as pharmaceutical and food companies, small and mid-sized enterprises (SMEs), start-up companies that use novel functional ingredients or local specialty foods, new entrants from other industries, and original equipment manufacturers (OEMs) [7,12].

A dietary supplement is a product marketed in dosage form (e.g., soft and hard capsules, tablets, powders and granules, liquids) that differs from regular food products and contains ingredients with nutritional or physiological effects. A dietary supplement contains one or more dietary ingredients such as a mineral, a vitamin, an amino acid, a medical herb, or other physiological ingredients for use to supplement the diet by increasing the total daily intake. Nutraceuticals are made up of these nutritional supplements and are used for health purposes other than nutrition. Although there is no common international definition of dietary supplement, it is generally positioned between pharmaceuticals and food under the laws and regulations of many countries [13]. For functional food products, most regulatory agencies require evidence to support their functionality, with regulations that ensure that the products entering the market can support these claims. Scholars have demonstrated the regulation of functional food products is necessary to protect both consumers’ safety and health and the industry from potential market failures [8,12,14,15,16].

Scholars of regulatory science and innovation management have explored the influence of technological and regulatory trajectories and the path-dependent or creative mechanism of the dietary supplement industry, and have found that the industry has been formed by the industrial integration of the pharmaceutical and food industries [17]. This example of industrial convergence highlights the theory that new industry sectors can form from the fusion of knowledge, technology, and businesses from diverse industries [18,19]. According to Bröring et al., in the case of dietary supplements, the pharmaceutical industry introduced technical competencies, whereas the food industry focused on marketing competencies, thus demonstrating the importance of collaborative research and development between the pharmaceutical and the food industries to achieve innovation [20,21].

### 2.2. Issues Surrounding Functional Foods

Chauhan et al. noted that healthy market competition requires appropriate regulations for product health claims [15]. Similarly, Akerlof observed that, without regulation, the information asymmetry between consumers and producers regarding functional foods could result in unfair competition [22]. When appropriately implemented, regulation ensures public health benefits and the safety of consumers. Similarly, labeling for dietary supplements and conventional foods protects consumers by enabling them to identify early signals that a product may present safety and health risks, thus ensuring product quality. Nasri et al. pointed out that, in the functional foods product segment, a market latecomer may develop and produce similar functional foods at a low cost, because it is difficult to protect food ingredients by patents, differing from pharmaceutical ingredients, which have patent protection [23]. This effect could prevent existing manufacturers from allocating resources for research on new functionalities of food. Therefore, according to Hobbs et al., restrictions for labeling health claims could suppress so-called free riders—those who benefit from competitors’ efforts and the lack of regulation—and promote innovation [14]. Conversely, overly strict regulations can hinder market competition. Excessive regulation adoption costs can reduce the efficiency of development and production and increase the cost of functional foods and dietary supplements.

Innovation is the application of ideas that result in new technology development and product introduction; it requires significant investment in research and development (R&D). The R&D investment of the food industry is lower than that of the pharmaceutical industry; this is a particular issue in the promotion of innovation in the food industry. To compensate for the shortage of resources for R&D efforts in creating functional food products, scholars have suggested the possibilities of a type of open-source development based on partnerships with universities and of open innovation based on sharing knowledge and information from cooperative networks of external partners [24,25].

Thus, the knowledge and technology resources of the food and pharmaceutical fields are integrated, and this industry–academia collaboration results in open innovation in the functional foods sector, and regulation—which has the dual role of promoting innovation and impeding innovation—controls this wide variety of actors [20,21,25,26].

### 2.3. The Health Food System and Regulatory Reform in Japan

The Japanese functional foods sector has been steadily growing; the current market volume is USD 20 billion per year. The Japanese regulatory system for foods with health claims (FHC) includes foods for specified health uses (FOSHU), foods with nutrient function claims (FNFC), and the newest category of foods with function claims (FFC). The FNFC category is a self-certified system, and the labeling mainly addresses conventional nutrients such as vitamins and minerals. The FOSHU and FFC systems are systems for labeling the health claims of food ingredients beyond these conventional nutrients.

FOSHU includes foods approved by Japan’s Consumer Affairs Agency (CAA) and includes manufactures’ labeling of food nutrients based on safety and efficacy evaluations supported by clinical trials [27]. Initiated in 1991, the FOSHU system allowed the labeling of foods to help consumers take a proactive approach to their health care. The FOSHU market has grown to over JPY 600 billion, accounting for approximately one-third of the Japanese health food market. However, the disadvantages of the FOSHU system include high costs and risks for manufacturers in the process of developing and bringing a product to market, with costly R&D and clinical trials and lengthy approval time [27].

The FFC system includes functional foods with information supporting the safety and effectiveness of the product submitted to, but not individually pre-approved by, the Secretary General of the CAA prior to product marketing. Thus, although the labeling of these foods’ function claims is based on scientific evidence, accuracy is the responsibility of food business operators [28,29,30,31,32]. Introduced in 2015, the less rigid FFC system was intended to stimulate the health food market through deregulation and result in food products that could potentially promote health, mitigate lifestyle-related diseases, and reduce health care costs for consumers. The clear labeling of nutritional or health information is intended to facilitate consumers’ ability to take a proactive approach to their health care and make more informed choices.

From the viewpoint of the health food industry, deregulation was intended to reduce companies’ costs and risks of product development [27,33]. In the FFC system, the Japanese government does not evaluate the safety and effectiveness of function claims, and thus has reduced the cost of the process by adopting a notification system with the responsibility of the operator as part of the administrative procedure. Thus, the FFC system not only provides more information about functional food products to consumers but also helps small companies develop functional foods [28,33,34]. The FFC system accelerated the entry of new competitors into the market, with the goal of increased market growth. Under the FFC system, the government certified many product health claims—including those related to eyes, joints, mental stress, cognitive function, sleep, physical fatigue, and obesity—that had not previously been approved under the FOSHU system [33].

**Figure 1:**
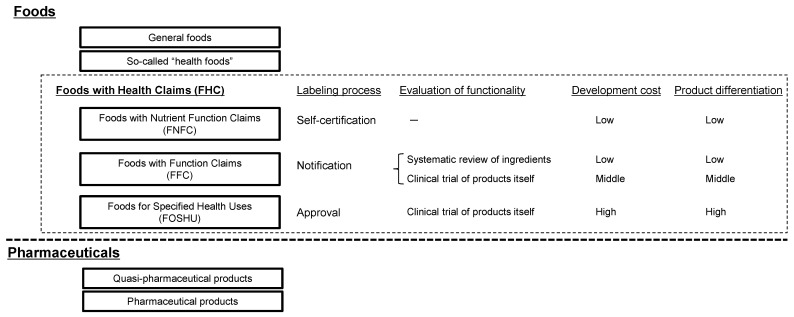
Food with health claims (FHC) system in Japan.

There are two ways for companies to evaluate the functionality of a product. One is using a systematic review (SR) of scholarly papers about clinical trials (CTs) of product ingredients, and the other is using a CT of the product itself (Figure 1) [29]. An SR allows the use of external knowledge to evaluate product claims instead of CTs conducted by the manufacturing companies themselves, and FFC products evaluated using the SR route may be developed at a lower cost than those evaluated using the CT route [30,33]. However, if a number of similar FFC products based on the same SR are introduced to the market by multiple companies, companies following the SR route for the evaluation of their FFC products may find it difficult to establish a competitive advantage. In contrast, FFC products evaluated using the CT route are more costly than those evaluated using the SR route, because the CTs for those products evaluated through the SR route are basically conducted in-house.

Foods are multi-ingredient and have moderate multi-functionality [28]. By combining product ingredients, making products multifunctional, and developing new functionality, manufacturers can develop innovative products and can potentially develop an original FFC product in-house by formulating and conducting CTs themselves, thus establishing product differentiation.

### 2.4. Research Objectives and Hypotheses

In this study, we empirically explored how the regulatory environment affected the innovation process in Japan during the transition of the regulatory approval process for functional foods.

Specifically, legislation for foods with health claims (FHC) expanded from only allowing function claims on food labels for foods for specified health uses (FOSHU) and foods with nutrient function claims (FNFC) to including a new type of FHC, the less stringent regime for foods with function claims (FFC). In particular, we examined the positive impact of the regulatory transition on industry and firm performance. We posit that the study of the Japanese FFC system is relevant to the discussion of the achievement of optimal regulation in the health-care sector, the issues of innovation promotion and inhibition in a regulatory environment, and how regulatory health-care reform—when properly implemented—can stimulate innovation in technology and the delivery of health care. We consider the timeframe for observation, seven years after the launch of the FFC system in 2015, sufficient to observe companies’ adaptation strategies for deregulation and their consequences.

We examined the influence of Japan’s FFC system on companies in the dietary supplement industry. One of the policy intentions for the introduction of the FFC system was to facilitate companies’ ability to develop and bring functional foods to the market, and to stimulate the health food industry by lowering the costs and risks of product development. This study set forth the following hypothesis:

**H_1_:**  *The introduction of the FFC system has led to the market entry of a diverse range of companies*.

In the FFC system, a variety of health claims emerged, including those that had not previously existed in the FOSHU system as well as multi-health claims. Some FFC products label multi-health claims relating to several functions, such as sleep and mental stress, gut condition and obesity, and joints and muscles. As a result, companies were able to develop FFC products with novel value, and the system provided companies new competitive opportunities. This study set forth the following hypothesis:

**H_2_:**  *Companies that use the FFC system perform better than companies that do not use the FFC system*.

The FFC system has two ways to evaluate the functionality of functional products. The SR route enables manufactures to develop FFC products at a low cost, efficiently, and rapidly by using external knowledge through SRs. The CT route enables manufactures to develop new, differentiated FFC products using in-house CTs. Based on these considerations, we hypothesized that the method of assessing product functionality (i.e., internal or external company test type) influences functional product sales. As one of the constitutive factors, we analyzed the approaches to assessing product functionality and product characteristics. The in-house testing type refers to an approach in which the company conducts its own CTs to substantiate the evidence of its product claims (i.e., the CT route), whereas the external testing type is based on an approach in which the company uses the existing evidence base documented by previous studies (i.e., the SR route). This study set forth the following hypothesis:

**H_3_:**  *Products evaluated by in-house testing have a higher market value than those evaluated by external testing*.

To verify H_1_, we analyzed the size and attributes of companies that provided the CAA notification of their FFC evidence (Analysis 1). To verify H_2_, we analyzed the sales and growth rate of the supplement business with and without the use of the FFC system (Analysis 2). To verify H_3_, we analyzed the product attributes and sales of FFC products (Analysis 3).

## 3. Materials and Methods

### 3.1. Analysis 1: Sales and Properties of Companies Submitting Foods with Function Claims (FFC)

Data were collected for 169 companies in the Japanese dietary supplement industry in 2019 [35,36]. The variables included the following:

- Corporate revenue;
- Main business of the company (pharmaceutical, food, retail, functional materials, supplement OEM, and other categories);
- Whether or not the company had a track record of handling FOSHU (food, beverages, or supplements) [37].

### 3.2. Analysis 2: Performance and Product Characteristics of Top Companies

First, the top 30 dietary supplement manufacturers were identified by aggregating product sales in an industry information data book [38]. Sales of these companies’ dietary supplements totaled JPY 590.7 billion per year, covering 61% of the Japanese dietary supplement market. Next, the dataset of 27 companies (excluding three companies that could not obtain sales data in 2015) was created by composing (a) the CAA’s database of FFC [35], and (b) relevant market research data [38]. For the 15 companies selling FFC products, four variables (based on the FFC product data set described in Analysis 3, showing their FFC product properties) were added. The variables included the following:

- Sales of dietary supplements in 2015 (when the FFC system started) and 2020;
- FOSHU dummy (if the company sells FOSHU, whether food, beverages, or dietary supplements; dummy is 1) [37];
- Indices of the properties of FFC products of each company (sales weighted by average number of product materials; sales weighted by average number of product functions; sales by weighted new function rate; and sales weighted by in-house CT rate).

### 3.3. Analysis 3: Product Attributes and Sales of Foods with Function Claims (FFC)

The product data set for 74 FFC products (sold by 15 companies) was created by composing (a) the CAA’s database of FFC [35] and (b) market research data [38]. The product variables included the following:

- Sales figures;
- Product properties, including the number of functional materials; the number of functions; whether the function is new or not; the number of papers on which the functionality is based; and the year of publication of the paper;
- CT implementation body (in-house trial type (in-house CT) or external trial type (External SR)), returning to product CTs and SR papers to verify whether they were in-house CTs or external SRs, categorizing products into the following two categories: In-house test type: products for which clinical trials are being conducted in-house; External test type: products that have not undergone clinical trials in-house;
- Release year (in addition to the year of notification of FFC, if the same product was marketed as a health food before this time, we included the year of its release).

Statistical analyses were performed using R statistical software (version 3.4.1, R Foundation, Vienna, Austria, 2017).

## 4. Results

### 4.1. Analysis 1: Revenue and Properties of Companies Submitting FFC

Table 1 provides the descriptive statistics of the 169 companies submitting dietary supplement-type FFC products, comparing the revenue of the companies using both the FOSHU and FFC system (i.e., the existing companies) and that of companies entering the new FFC system (i.e., the newly entering companies). The Wilcoxon rank sum test was employed to compare the revenue. The test revealed that the existing companies have significantly more revenue (*p* < 0.01). The 121 companies that newly entered the FFC claim system were outnumbered by the 48 existing companies who had FOSHU (either food, beverage, or dietary supplements).

The corporate characteristics of entering companies differed from those of existing ones. Although the existing company group consisted of 22 food companies (46%) and 11 pharmaceutical companies (23%), accounting for two-thirds of the total, the entering company group consisted of 17 food companies (14%) and six pharmaceuticals companies (5%). Conversely, in the entering company group, retailers (68 companies, 56%) and raw material manufacturers (14 companies, 12%) were more than those in the existing company group.

Table 2 shows the revenue of retail companies and that of non-retail companies in the entering group. The Wilcoxon rank sum test between them revealed that revenue of retail companies is significantly less than that of non-retail (*p* < 0.01).

Among the 169 companies, no companies submitted all FFC products via only the CT route; 132 companies (78%; SR only companies) had only SR FFC products, and 37 companies (22%; SR and CT companies) had both CT FFC products and SR FFC products. The number of SR FFC products was 652, which accounted for 89% of the total of 731 FFC products. Table 3 shows the results of the comparison between the SR group and the SR and CT company group. The Wilcoxon’s rank sum test revealed that the former group companies had lower revenue than the latter. This suggests that small companies, in particular, used the SR route.

In summary, under the FFC system, many small-scale companies, especially retailers, have entered the market of dietary supplements with health claims, showing the diversity of companies in the market. This supports H_1_.

### 4.2. Analysis 2: Sales and Growth Rate of the Dietary Supplement Business with and without the Use of Foods with Function Claims

Supplementary Figure S1 shows the details of the top 27 dietary supplement companies. Table 4 shows the dietary supplement sales and the compound annual growth rate (CAGR) for 12 companies without FFC products (non-FFC company) and 15 companies with FFC products (FFC company). Wilcoxon’s rank sum test showed that the CAGR of FFC companies was larger than that of non-FFC companies (*p* = 0.01). This suggests that the use of the FFC system is linked to corporate growth. Therefore, H_2_ was supported.

### 4.3. Analysis 3: Product Properties and Sales of Foods with Function Claims (FFC)

Table 5 shows the classification of 74 FFC products. Among them, 12 products were shown with functionality by product CT, 57 products were submitted via the ingredient SR route, and five products demonstrated functionalities by a combination of product CTs and ingredient SRs. These were classified into in-house study types and external study types, according to the entity conducting the clinical study.

Among the 12 “CT type” products, two products were classified as “External CT” because those CTs were conducted by raw material manufacturers rather than by FFC product manufacturers. That is, from the perspective of FFC product manufacturers, these products were developed efficiently using external product clinical tests and formulations.

Among the 57 ingredient SR type products, 10 products were categorized as in-house CTs, because their systematic review included article(s) reporting CTs conducted by the FFC product manufacturers themselves. As a result, 74 FFC products were divided into 25 in-house test products and 49 external test products. Supplementary Figure S2 shows a detailed list of the 74 FFC products.

Table 6 shows the comparison of sales, number of CT papers, year of publication, number of materials, and number of functions between in-house test type products (n = 25) and external test type products (n = 49). Table 7 shows the correlation coefficients between indicators for 74 FFC products. Table 8 shows the results of multiple regression analysis of sales with four independent variables (number of materials, number of functions, new function as a dummy variable, and in-house as the dummy variable).

In the external trial type, the average number of papers used for efficacy evaluation was 5.1 papers, published from the 1980s to the 2010s (the median year was 2010). In the in-house trial type, the average number of papers was 2.8, and the median year of publication was 2015. The Wilcoxon’s rank sum test revealed that the external trial type evaluated their efficacy by more papers published over a longer period.

The correlation coefficients in Table 7 reveals that number of materials, number of functions and in-house CT dummy are correlated with each other (correlation coefficients: 0.25 to 0.39, *p* < 0.05). Because the maximum of all correlation coefficients is r = 0.54 (between Sales and in-house CT dummy, *p* < 0.01), showing loose positive relationships, there is little concern about multicollinearity. Multiple regression analysis of sales including all variables was run.

The results of multiple regression analysis showed that the coefficient for the in-house CT dummy was 26.8 (95% confidence interval (CI) [15.9, 37.6], *p* = 0.00001). This result revealed that the in-house test type had higher sales than the external CT type. The coefficient for number of materials was 7.6 (95% CI [3.5, 11.7], *p* = 0.0004). The coefficient for new function dummy was 10.2 (95% CI [0.4, 20.1], *p* = 0.04). This model confirms that in-house CT, large volume of materials, and new functions could increase sales of FFC products. Thus, H_3_ was supported. In-house test-type products combine functional materials, conduct CTs, create knowledge in-house, and differentiate themselves as multi-functional products, resulting in high sales.

## 5. Discussion

### 5.1. Assessment of the FFC System

Functional foods are developed through a long and highly uncertain process, which includes gathering knowledge about diseases and nutrition, obtaining evidence, and responding to regulations [25]. The FFC system, which consists of notifications by an SR of ingredients or clinical trial for individual products, is a deregulation of the FOSHU system, whose products are admitted based on clinical trial evidence. This deregulation has led to an increase in the number of companies entering the system, especially small retailers. This is expected to lead to the provision of products that better meet consumer needs, allowing consumers to proactively manage their healthcare.

The FFC system provides a variety of product development strategies for manufacturing companies. In notification using SR, the company supplements its own research and development resources with accumulated external knowledge to commercialize its product efficiently. From an industry perspective, collaboration with academia and government could reduce companies’ R&D costs and risks [39]. We posit that the creation and systematization of knowledge by the public sector will be useful. Some companies have launched differentiated and competitive FFC products to realize their corporate growth by adapting to the new FFC system. As it is not easy for follower companies to use the evidence of clinical trials conducted by leading companies on their unique formulations, the latter can increase the competitiveness of their products with their uniqueness and differentiation. These R&D activities are expected to lead to market introduction and popularization of new products, which in turn can lead to innovation.

The FFC system is a case of a regulatory system (re)design that promotes innovation by adjusting and optimizing the level of regulation. To promote health, mitigate lifestyle-related chronic diseases, and reduce healthcare costs, non-medical products and services that allow consumers to proactively manage their health care are required. This suggests the possibility of creating innovation through deregulation and open innovation using a different approach from that of the medical industry, which typically makes significant investments in R&D.

However, there are also some concerns about the current FFC system. Some health benefits are unsuitable for functional claims, which limits product development under the FFC system. Some studies indicate that SR used in FFC is unreliable [29,30]. In addition, the combination of multiple ingredients has a possibility of unexpected effects in terms of safety due to the interaction of the ingredients. To further the research on the functions and effects of ingredients, future scholars, practitioners, and policymakers should engage in transdisciplinary R&D.

### 5.2. Prospects for Japanese and International Functional Food Systems

The number of FFC products continues to grow steadily; however, there are also systemic distortions of functional foods as a whole. In particular, there are many companies that produce so-called “health foods” without using the FHC system. The FFC system has facilitated company entry, but utilization is left to the company’s discretion, and loopholes remain, particularly for those that are prone to opportunistic behavior. It should also be noted that these is an overlap of FOSHU and FFC in the current regulation system. The FFC system allows companies to label health claims that are similar to those used for FOSHU (and even those not available for FOSHU) at a lower cost. FFC with similar health claims as FOSHU may confuse consumers and would be a threat to companies selling FOSHU. The complexity of the Japanese functional food regulations shown in Figure 1 is attributable to a repeatedly revised and expanded system [7]. Redesigning the Japanese regulation framework would be difficult because of the historical path-dependency of the system, even though reorganization of the regulation system (such as repositioning FOSHU) would be desirable.

Looking at another case, in the U.S., which is the largest market for supplements, the Dietary Supplement Health and Education Act (DSHEA) allowed dietary supplements to label functional claims as of 1994 [7,9]. Since then, the dietary supplement market in the U.S. has expanded significantly. Labels related to functionality increase consumer willingness to purchase and stimulate the market. However, the DSHEA, applicable only to dietary supplements, does not allow clinical trials on individual products. Hence, some of the sales factors revealed in this study, such as in-house clinical trials, would not be applicable to the U.S. market.

FFC is a unique system containing a double track of notification by SR of ingredients and clinical trial for individual products, leading to reduced administrative costs and promotion of product development competition among companies. It is possible that, in regulatory systems other than that of Japan, system revisions that allow notification by product clinical trials would bring market expansion through industrial revitalization and new product development. Regulations for functional foods vary from country to country. In addition to the U.S., according to Singapore’s regulations, which require notification, clinical trials for individual products are not permitted; however, regulations in some countries such as South Korea and Taiwan require approval for individual products [34].

Conversely, the Japanese system is not sufficiently harmonized internationally. Compared to other systems, the Japanese system has some unique practices, such as voluntary good manufacturing practice for quality control [7,34]. The lack of global regulatory harmonization could bar foreign companies from entering the Japanese market; it could also reduce product variety, limiting Japanese consumers’ access to and choice of products, including those from overseas. Moreover, Japanese companies incur additional costs to comply with foreign regulations when exporting their products overseas.

### 5.3. Limitations and Future Perspectives

This study has several limitations. It employed the cross-sectional data of 74 FFC products from 15 companies in Japan. As such, it was limited to a specific period or regional context. To obtain deeper insights into the relationship between innovation and regulation in the functional foods market, future scholars should conduct a time-course observation and analysis of these cases. In addition, the present study’s analysis was conducted using externally observable indicators, while product sales are influenced by many kinds of factors such as type of functionalities, brand image, product price, product channel, or promotions. Further studies are needed to address these points.

## 6. Conclusions

In this study, we empirically explored how the regulatory environment affected the innovation process in Japan during the transition of the regulatory approval process for functional foods. The Japanese FFC system allowed the entry of several companies, mainly small retailers, into the Japanese FHC market. This deregulation contributed to an increase in the diverse range of companies entering the market and broadening its base. The relatively high compound annual growth rates of companies utilizing the FFC system suggest that this scheme also contributed to the growth of the companies.

Under FFC with its notification system, it is difficult to achieve product competitiveness by adapting to regulations, compared to the situation under FOSHU’s approval system. The FFC system’s double-track notification routes diversify corporate strategic options and make companies compete based on their respective strategies. Small companies tend to use SR, based on external knowledge, to reduce development costs. To increase product value in the market, it is necessary to strengthen the required efforts, including the development of multiple-materials formulas and in-house testing processes. The FFC system, which is unique to Japan, promotes competition among diverse companies by encouraging them to develop competitive products to expand the market. However, the Japanese functional foods system has become complex and does not harmonize with the other international regulatory systems.

Finally, we propose two suggestions for the Japanese functional foods system. The system, including FOSHU and so-called health foods, should be totally reorganized. International regulatory harmonization should be promoted to improve consumer accessibility. We expect that these findings on the relationship between regulation and innovation will provide useful implications for scholars, practitioners, and policymakers for its optimization in the dietary supplements market.

## Figures and Tables

**Table 1:** Number of companies by attribute and corporate revenue.

Number of Companies							Revenue (100 Million Yen)				
	Pharm.	Food	Retail	Material	OEM	Other	Minimum	Maximum	Mean	S.D.	
Existing Company (n = 48)	11 (23%)	22 (46%)	9 (19%)	2 (4%)	2 (4%)	2 (4%)	5	14,894	2495	3485	*p* = 0.000
Entering Company (n = 121)	6 (5%)	17 (14%)	68 (56%)	14 (12%)	7 (6%)	9 (7%)	0.2	19,154	700	2376	

Note: The existing companies group consists of those that use both the FOSHU and FFC systems. The entering companies group consists of those entering the new FFC system. The six categories are representative of the main business domain of each company: pharmaceuticals, food, retail, functional materials, supplement OEM, and other. Revenue is corporate revenue of the manufacturing company (refer to Section 3).

**Table 2:** Corporate revenue of retailers and non-retailers among entering company group.

Variables	Retail Company (n = 68)		Other Than Retail (n = 53)		*p*-Value
	Mean	S.D.	Mean	S.D.	
Revenue (100 million yen)	368	1481	1126	3123	0.00

Wilcoxon rank sum test. Note: Revenue is the corporate revenue of the manufacturing company (refer to Section 3).

**Table 3:** Corporate revenue of 169 companies by notification route.

Variables	SR Only (n = 132)		SR & CT (n = 37)		*p*-Value
	Mean	S.D.	Mean	S.D.	
Revenue (100 million yen)	1123	2732	1520	3233	0.01

Wilcoxon rank sum test. Note: SR = systematic review; CT = clinical trial. Revenue is the corporate revenue of the manufacturing company (refer to Section 3).

**Table 4:** Sales and CAGR of 12 companies without food with function claims (FFC) and 15 companies with FFC.

Variables	Non-FFC (n = 12)		FFC (n = 15)		*p*-Value
	Mean	S.D.	Mean	S.D.	
Sales (100 million yen)	171.4	119.2	229.8	195.3	0.24
CAGR	−0.022	0.045	0.061	0.100	0.01

Wilcoxon rank sum test. Note: FFC means foods with function claims. Sales are those of dietary supplements in 2015 (refer to Section 3, Materials and Methods). CAGR (compound annual growth rate) was calculated from sales in 2015 and 2020.

**Table 5:** Notification routes for foods with function claims (FFC) and the number of products by clinical trial implementation body (n = 74).

	In-House CT (n = 25)	External CT (n = 49)
Product CT (n = 12)	10	2
Ingredient SR (n = 57)	10	47
Hybrid (n = 5)	5	

Note: SR = systematic review; CT = clinical trial.

**Table 6:** Inter-group testing between in-house and external test types (sales, number of clinical trials (CT) papers, year of publication, number of materials, number of functions).

Variables	In-House CT (n = 25)		External CT (n = 49)		*p*-Value
	Mean	S.D.	Mean	S.D.	
Sales (100 million yen)	3.5	3.7	0.5	0.8	0.00002
Number of Articles about CT	2.8	3.0	5.1	3.8	0.001
Published Year (Median)	2014.9	3.9	2009.7	5.5	0.00007
Number of Materials	2.0	1.3	1.3	1.2	0.002
Number of Functions	1.7	0.9	1.1	0.4	0.002

Wilcoxon rank sum test. Note: CT = clinical trial. Refer to Section 3 for explanations of the variables.

**Table 7:** Correlation coefficients between indicators for 74 foods with function claims (FFC) products.

	Sales		Number of Materials		Number of Functions		New Functions		In-House CT Dummy
Sales	1								
Number of Materials	0.46	**	1						
Number of Functions	0.22		0.37	**	1				
New Functions	0.25	*	0.12		0.12		1		
In-house CT dummy	0.54	**	0.25	*	0.39	**	0.07		1

Note: *p*-values of correlation analysis are shown by * and ** (*: *p* < 0.05; **: *p* < 0.01). CT = clinical trial. Refer to Section 3 for explanations of the variables.

**Table 8:** Multiple regression analysis of product sales.

Variables	Coefficient t	Std. Error	95% Confidence Interval		t Value	*p*-Value
			Lower	Upper		
Constant	−5.2	6.0	−17.1	6.7	−0.9	0.39
Number of Materials	7.6	2.1	3.5	11.7	3.7	0.0004
Number of Functions	−4.9	4.1	−13.0	3.2	−1.2	0.23
New Functions	10.2	4.9	0.4	20.1	2.1	0.04
In-house CT dummy	26.8	5.4	15.9	37.6	4.9	0.00001

Note: CT = clinical trial. Refer to Section 3 for explanations of the variables.

## Data Availability

The data presented in this study are openly available in References [35,36,37,38].

## Supplementary Materials

The following supporting information can be downloaded at: https://www.mdpi.com/article/10.3390/nu15020476/s1, Figure S1: Dataset of the 27 target companies; Figure S2: List of products (n = 74).
