# Supplementary material for: Optimizing the Relationship between Regulation and Innovation in Dietary Supplements: A Case Study of Food with Function Claims in Japan
Source: Nutrients. 2023 Jan 16;15(2):476. doi: 10.3390/nu15020476 (PMC9866933; doi:10.3390/nu15020476)
Supplement: Supplementary file 1 [file nutrients-15-00476-s001.zip › nutrients-2128769-supplementary.pdf]

Supplementary Materials

ID	FFC	Existing Company	Sales	CAGR	Sales Rate of FFC	Properties of FFC products (Sales Weighted Average)					
						No. of FFC	in-house CT rate	Ave. No. of Ingredients	Weighted Ave. No. of Materials	Weighted Ave. No. of Functions	New Function Adopsion Rate
1			83.3	4.2%	35%	3	100%	3.4	3.1	1.3	100%
2	✓	✓	45.8	1.5%	15%	10	0%	1.5	1.1	1.3	87%
3			44.6	-2.7%	0%						
4	✓		43.1	9.1%	58%	16	66%	3.2	2.7	2.3	59%
5			37.5	1.4%	0%						
6	✓	✓	23.6	4.2%	6%	1	100%	3.0	3.0	1.0	0%
7	✓		22.7	-6.7%	0%	0					
8	✓		22.2	16.7%	14%	12	59%	1.3	1.0	1.6	48%
9			21.9	1.1%	0%						
10	✓	✓	17.3	12.9%	12%	4	35%	1.9	1.5	1.0	61%
11	✓		15.7	9.9%	51%	3	93%	2.0	1.9	1.0	100%
12			15.7	2.5%	0%						
13	✓	✓	15.2	4.5%	56%	2	58%	1.0	4.3	1.0	100%
14	✓		14.8	0.0%	0%	1	0%	1.0	1.0	1.0	0%
15	✓	✓	13.7	-10.4%	4%	2	26%	1.0	1.0	1.0	100%
16	✓	✓	12.6	-1.0%	4%	7	0%	1.2	1.0	1.0	30%
17			11.9	3.7%	0%						
18	✓		11.2	-4.0%	87%	2	100%	1.0	1.0	1.0	100%
19			11.0	-3.3%	0%						
20	✓	✓	9.6	9.1%	40%	5	16%	1.0	1.0	1.3	26%
21			9.2	-9.4%	0%						
22	✓		8.9	34.3%	100%	5	98%	4.0	4.0	3.1	94%
23			8.7	-6.7%	0%						
24			7.8	-4.1%	0%						
25	✓	✓	7.6	0.3%	85%	1	100%	1.0	1.0	1.0	100%
26			7.5	4.4%	0%						
27			7.2	-6.2%	0%						

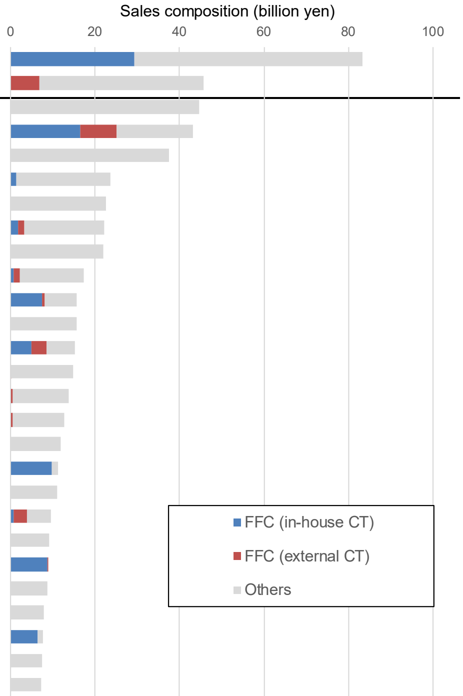

Supplementary Figure S1. Dataset of the 27 target companies.

ID	Type	Evaluation: CT	Evaluation: SR	Sales	No. of Materials	No. of Functions	New Function	No. of articles
1	in-house	✓		9.4	1	1	✓	1
2	↓	✓		5.0	1	1	✓	1
3	↓	✓		6.5	1	1	✓	1
4	↓	✓		0.1	1	1	✓	1
5	↓	✓		0.6	1	1		1
6	↓		✓	9.0	3	1	✓	1
7	↓		✓	0.1	1	1	✓	5
8	↓	✓	✓	0.7	1	1	✓	3
9	↓		✓	9.1	2	2	✓	2
10	↓	✓		0.4	1	1	✓	1
11	↓		✓	11.3	4	1	✓	2
12	↓	✓	✓	6.2	4	3	✓	5
13	↓	✓		7.5	2	1	✓	1
14	↓	✓	✓	2.0	5	4	✓	6
15	↓	✓		3.2	3	2		1
16	↓		✓	1.3	3	1		2
17	↓	✓		0.7	2	2	✓	2
18	↓		✓	0.2	1	1		12
19	↓	✓		0.3	1	3	✓	1
20	↓	✓	✓	5.6	4	4		2
21	↓	✓	✓	0.7	1	2		1
22	↓		✓	0.2	1	1		2
23	↓		✓	0.5	1	2		3
24	↓		✓	6.9	3	2	✓	2
25	↓		✓	0.4	1	2	✓	12
26	External		✓	0.1	1	1		5
27	↓		✓	0.3	1	1		6
28	↓		✓	0.7	1	1		5
29	↓		✓	0.4	1	1	✓	12
30	↓		✓	0.1	1	1		6
31	↓		✓	0.2	1	2	✓	5
32	↓		✓	0.0	1	2	✓	5
33	↓		✓	0.6	3	1	✓	5
34	↓		✓	0.5	1	1	✓	6
62	↓		✓	3.6	9	1	✓	2
35	↓		✓	0.2	1	1	✓	3
36	↓		✓	0.0	1	2	✓	5
37	↓		✓	0.5	1	1		2
38	↓		✓	0.5	1	1	✓	4
39	↓		✓	0.2	1	1	✓	7
40	↓		✓	0.1	1	1	✓	17
41	↓		✓	0.3	1	1		5
42	↓		✓	0.1	1	1		7
43	↓		✓	0.6	1	1	✓	4
44	↓		✓	0.2	1	1	✓	6
45	↓		✓	0.5	1	1	✓	3
46	↓		✓	0.9	1	1		1
47	↓		✓	0.2	1	1		2
48	↓		✓	0.4	1	1	✓	1
49	↓		✓	0.3	1	1		4
50	↓		✓	0.0	1	1		3
51	↓		✓	0.8	1	1		2
52	↓	✓		0.3	1	1	✓	1
53	↓		✓	0.1	1	1	✓	2
54	↓		✓	2.0	1	2	✓	12
55	↓		✓	0.6	2	1		12
56	↓		✓	0.4	1	1	✓	5
57	↓		✓	0.0	1	1	✓	2
58	↓		✓	0.1	1	1		14
59	↓		✓	0.7	2	1		11
60	↓		✓	0.0	2	2	✓	2
61	↓		✓	1.4	1	1	✓	5
63	↓	✓		0.1	1	1	✓	1
64	↓		✓	0.6	1	1	✓	2
65	↓		✓	4.4	2	2	✓	14
66	↓		✓	0.0	1	1		7
67	↓		✓	0.3	1	1		2
68	↓		✓	0.3	1	1		7
69	↓		✓	0.1	1	1		2
70	↓		✓	1.0	1	2	✓	5
71	↓		✓	0.4	1	1	✓	6
72	↓		✓	1.6	1	1	✓	2
73	↓		✓	0.1	2	1	✓	1
74	↓		✓	0.1	1	1	✓	1

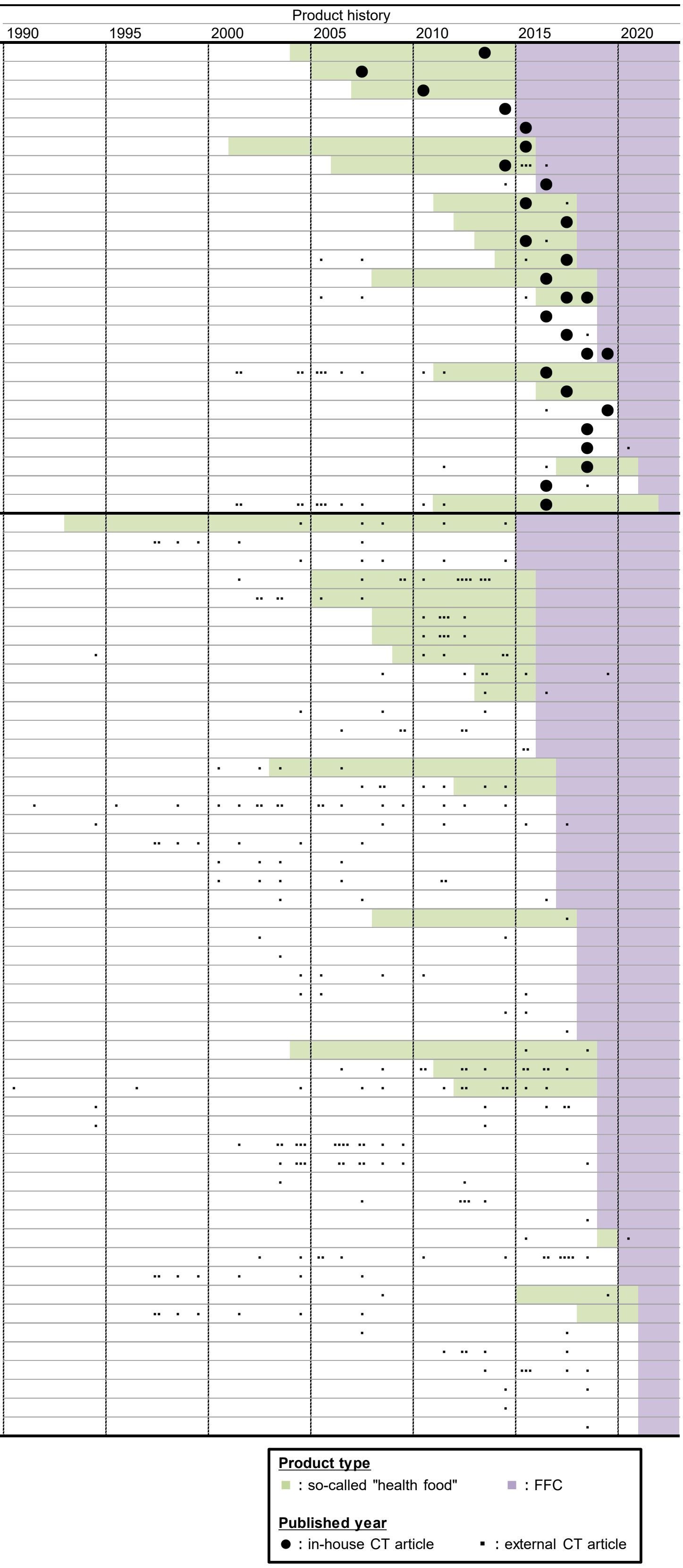

Supplementary Figure S2. List of products (n = 74).
